# Supplementary material for: Identification and functional analysis of non-coding regulatory small RNA FenSr3 in Bacillus amyloliquefaciens LPB-18
Source: PeerJ. 2023 May 15;11:e15236. doi: 10.7717/peerj.15236 (PMC10194069; doi:10.7717/peerj.15236)
Supplement: Supplemental Information 4 [file peerj-11-15236-s004.zip › KO/CK-vs-T1_map/map00720.html]

KEGG PATHWAY: Carbon fixation pathways in prokaryotes - Reference pathway


|  |  |
| --- | --- |
| **Carbon fixation pathways in prokaryotes - Reference pathway** |  |

[
Pathway menu
| Organism menu
| Pathway entry
| Show description
| User data mapping
]

Carbon fixation is an important pathway for autotrophs living in various environments. Plants and cyanobacteria fix CO2 as organic compounds using solar energy mainly by the reductive pentose phosphate cycle (also called Calvin cycle, Calvin-Benson cycle, or Calvin-Benson-Bassham cycle) [MD:M00165]. There are, at least, five additional carbon fixation pathways known to exist in autotrophic bacteria and archaea, which differ in reducing compounds, energy source, and oxygen sensitivity of enzymes. (i) The reductive citric acid cycle (Arnon-Buchanan cycle) [MD:M00173] is found in microaerophiles and anaerobes, such as green sulfur bacteria. In one complete turn of this cycle, four molecules of CO2 are fixed by the enzymes that are sensitive to oxygen, resulting in the production of one molecule of oxaloacetate, which is itself an intermediate of the cycle. (ii) The reductive acetyl-CoA pathway (Wood-Ljungdahl pathway) [MD:M00377] is found in strictly anaerobic bacteria and archaea (Proteobacteria, Planctomycetes, Spirochaetes, and Euryarchaeota), some of which are methane-forming. A bifunctional enzyme, carbon monoxide dehydrogenase/acetyl-CoA synthase, catalyzes the reactions from CO2 to CO and from CO2 to a methyl group, and then to generate acetyl-CoA. (iii) The 3-hydroxypropionate bicycle [MD:M00376] is found in some green non-sulphur bacteria of the family Chloroflexaceae. In one complete turn of this bicycle, three molecules of bicarbonate are converted into one molecule of pyruvate. In addition, this bicycle provides the secondary benefit of useful intermediates for biosynthesis: acetyl-CoA, glyoxylate, and succinyl-CoA. (iv) The hydroxypropionate-hydroxybutyrate cycle [MD:M00375] is found in aerobic Crenarchaeota, Acidianus, Metallosphaera, and Sulfolobales. Some of the intermediates and the carboxylation reactions are the same as in the 3-hydroxypropionate bicycle. One complete turn of this cycle generates two molecules of acetyl-CoA, one of which is reutilized in the the cycle and the other is removed for cell material biosynthesis. (v) The dicarboxylate-hydroxybutyrate cycle [MD:M00374] was named after its intermediates: succinate (a kind of dicarboxylate) and hydroxybutyrate. This cycle has been found only in Ignicoccus hospitals, a strictly anaerobic hyperthermophilic archaea. Recent genome study suggests that this cycle may exist in Desulfurococcales (to which Ignicoccus belongs) and Thermoproteales (a taxon close to the origin of archaea). The first half of the cycle, from acetyl-CoA to succinate-CoA, corresponds to the reductive citric acid cycle and the latter half of the cycle, from succinate-CoA to two molecules of acetyl-CoA, corresponds to the hydroxypropionate-hydroxybutyrate cycle.

|  |  |  |
| --- | --- | --- |
| Reference pathway | 184% 150% 122% 100% 82% 67% 55% | 图片下载 |
